# Hip pathologies in mucopolysaccharidosis type III

**DOI:** 10.1186/s13018-021-02340-6

**Published:** 2021-03-19

**Authors:** Sandra Rafaela Breyer, Eik Vettorazzi, Leonie Schmitz, Amit Gulati, Katharina Maria von Cossel, Alexander Spiro, Martin Rupprecht, Ralf Stuecker, Nicole Maria Muschol

**Affiliations:** 1 Department of Pediatric Orthopedics, Children’s Hospital Altona, Bleickenallee 38, 22763 Hamburg, Germany; 2 Department of Orthopedics, University Medical Center Hamburg-Eppendorf, 20246 Hamburg, Germany; 3 International Center for Lysosomal Disorders, University Medical Center Hamburg-Eppendorf, 20246 Hamburg, Germany; 4 Department of Medical Biometry and Epidemiology, University Medical Center Hamburg-Eppendorf, 20246 Hamburg, Germany; 5 Department of Pediatrics, University Medical Center Hamburg-Eppendorf, 20246 Hamburg, Germany

**Keywords:** Mucopolysaccharidosis type III, Sanfilippo syndrome, MPS, Osteonecrosis, Hip dysplasia, Femoral head, Pain, Skeletal disease, Skeletal dysplasia, Dysostosis multiplex

## Abstract

**Background:**

Mucopolysaccharidosis type III (MPS III) comprises a group of rare lysosomal storage diseases. Although musculoskeletal symptoms are less pronounced than in other MPS subtypes, pathologies of hip and spine have been reported in MPS III patients. The purpose of this study was to describe hip pathologies and influencing parameters in MPS III patients.

**Methods:**

A retrospective chart review was performed for 101 MPS III patients. Thirty-two patients met the inclusion criteria of enzymatically or genetically confirmed diagnosis and anteroposterior radiograph of the hips. Modified Ficat classification, Wiberg’s center-edge angle, and Reimer’s migration percentage were measured.

**Results:**

The mean age at data assessment was 11.0 years (SD 5.7). Osteonecrosis of the femoral head was observed in 17/32 patients. No statistically significant association was found between these changes and age, sex, or MPS III subtype. Patients with a severe phenotype showed significantly higher rates of osteonecrosis (14/17) than patients with an intermediate phenotype. Hip dysplasia was present in 9/32 patients and was significantly associated with osteonecrosis of the femoral head (*p* = 0.04).

**Conclusions:**

The present study demonstrates a high rate of hip pathologies in MPS III patients. Hip dysplasia and severe phenotype were significantly correlated with osteonecrosis of the femoral head. Therefore, radiographs of the hips are highly recommended in baseline and follow-up assessments of MPS III patients.

**Trial registration:**

Retrospectively registered.

## Background

Mucopolysaccharidosis type III (MPS III, Sanfilippo syndrome) is the most common type of MPS and comprises a group of clinically indistinguishable lysosomal storage diseases. These rare, autosomal recessive disorders are caused by deficiency of one of four enzymes (subtypes A–D) involved in the degradation of heparan sulfate (HS), including heparan N-sulfatase (sulfamidase), α-N-acetylglucosaminidase (NAGLU), acetyl-coenzyme A α-glucosaminide-N-acetyltransferase, and N-acetylglucosamine-6-sulfatase [1]. MPS IIIA is the most common subtype in northern Europe, with an estimated incidence in Germany of 1 in 63,700 births [2].

The clinical manifestations and disease progression of the MPS III subtypes vary due to differences in residual enzyme activities caused by several mutations in the four affected genes. Patients with MPS III present with behavioral abnormalities, sleep disturbances, and delayed speech development in early childhood. As the disease progresses, deterioration of neurological functions as well as motor skills becomes evident, leading to severe dementia during childhood [3]. In addition, radiologic signs of skeletal dysostosis multiplex are observed [1, 4]. Although musculoskeletal pathologies are less severe in MPS III compared to other MPS types, spine pathologies and a high prevalence of femoral head necrosis have been reported [4, 5]. The cause of femoral head necrosis in MPS III patients has not yet been histopathologically evaluated. The underlying genetic disease involves accumulation of dermatan and keratin sulfate, which can affect bone development and metabolism by causing inflammation and consequent abnormal bone (re-)modeling; these changes may mimic classical osteonecrotic changes [6–8].

As clinical assessment of hip disease is often challenging due to the hyperactivity and limited communication skills of these patients, hip pathologies might be overlooked. The purpose of this study was to describe hip pathologies and influencing parameters in MPS III patients.

## Methods

A retrospective chart review of 101 MPS III patients treated in the interdisciplinary outpatient clinic was performed (Table 1). The inclusion criteria were a genetically or enzymatically confirmed diagnosis of MPS III and one anteroposterior (AP) radiograph of the hips. Two patients were excluded from the analysis: one had a previous bone marrow transplantation, and the other showed unilateral signs of osteonecrosis of the femoral head, which was graded as classical Perthes’ disease. Thirty-two patients met the inclusion criteria.

**Table 1:** Patient characteristics

Characteristic	Total	Necrosis of femoral head		Correlation to necrosis
		No	Yes	
	***n*** = 32	***n*** = 15 (46.9%)	***n*** = 17 (53.1%)	
Age (years)				
Mean ± SD	11.0 ± 5.7	11.1 ± 6.1	10.9 ± 5.4	
Range		3.3–27.0	3.4–23.6	
Sex—no. (%)				
Male	21 (65.6)	8 (38.1)	13 (61.9)	*p* = 0.266
Female	11 (34.4)	7 (63.6)	4 (36.4)	
MPS subtype—no. (%)				
IIIA	26 (81.2)	13 (50.0)	13 (50.0)	*p* = 0.795
IIIB	4 (12.5)	1 (25.0)	3 (75.0)	
IIIC	2 (6.2)	1 (50.0)	1 (50.0)	
Phenotype—no./total no.(%)				
Severe	23/30 (76.7)	9 (39.1)	14 (60.9)	*p* = 0.036*
Intermediate	7/30 (23.3)	6 (85.7)	1 (14.3)	
Dysplasia—no. (%)				
No	23 (71.9)	13 (56.5)	10 (43.5)	*p* = 0.04*
Yes	9 (28.1)	2 (22.2)	7 (77.8)	

*No*. number, *SD* standard deviation

*Significant

Due to the clinical variability of MPS III, patients were classified as severe, intermediate, or attenuated based on either the underlying mutation (from known genotype-phenotype correlations) or the 4-point scoring system (FPSS) if the mutation was unknown or has not yet been described [3, 9–12]. The FPSS is an instrument assessing the degree of developmental regression (motor function, speech abilities, and cognitive function) over the course of the disease, which enables a classification of the patients into severe, intermediate, and attenuated forms.

Anteroposterior radiographs of the hips were available for all 32 patients. Radiographs were performed in supine position with the hips in neutral rotation. Because of the patients’ hyperactivity, frog-leg lateral view radiographs were not routinely performed. For 16 patients, the radiological assessment was performed during a routine consultation as a baseline checkup without clinical signs. For the remaining 16 patients, pain and/or advanced difficulties in walking as well as asymmetry in posture were the indications for radiological evaluation. Radiological images were digitally transferred and evaluated by an experienced pediatric orthopedic surgeon using Centricity PACS Universal Viewer (Version 5.0, GE Healthcare, Little Chalfont, UK). An adaption of the modified classification system of Ficat for MPS III patients as published by de Ruijter was used to describe the severity of osteonecrosis of the femoral head (Table 2) [4].

**Table 2:** Adaption of the modified Ficat classification [4] and distribution in study group

Stage 1	Minor changes on radiograph	25%
Stage 2 A	Sclerosis or cysts of femoral head, diffuse porosis	25%
Stage 2 B	Crescentic subchondral line, flattening of the femoral head	28%
Stage 3	Broken contour of the head, normal joint space	16%
Stage 4	Collapse, flattened contour of the femoral head, decreased joint space, osteoarthritis	6%

Percentage distribution of single hips affected by osteonecrosis (*n* = 30)

**Fig. 1:**
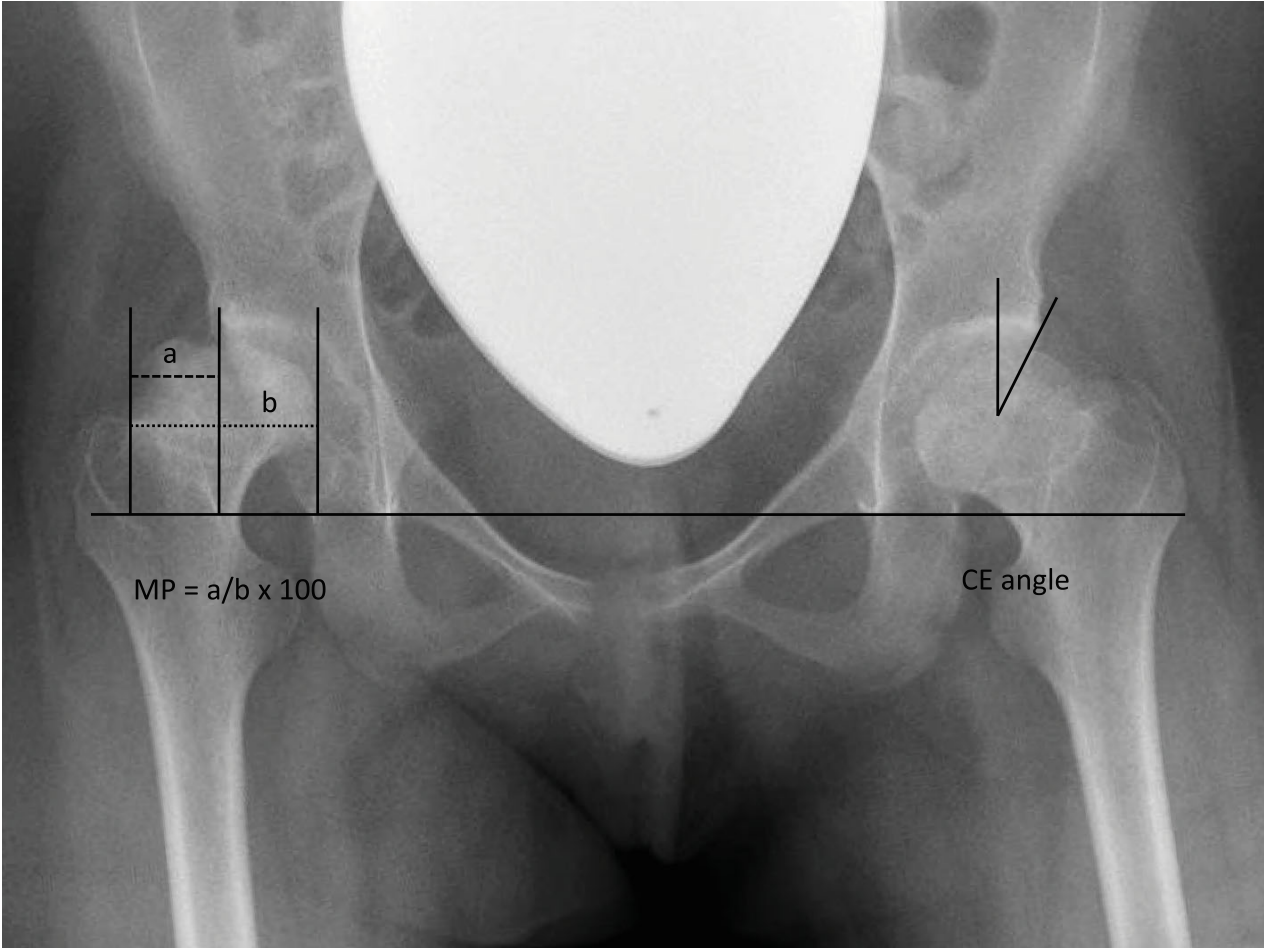
Radiograph of a 15-year-old severely affected female patient demonstrating measurement of the Reimers percentage (MP, right hip) and Wiberg's center-edge angle (CE, left hip).

Acetabular coverage was analyzed by measuring Wiberg’s center-edge (CE) angle [13], and Reimer’s migration percentage (MP) was used as an index of hip migration (Fig. 1) [14]. Dysplasia of the hip was classified using the Severin classification; hips classified as ≥ group III were defined as dysplastic [15]. The femoral neck-shaft angle was measured only on the AP view of the hip owing to missing lateral views. In addition, classification of the proximal femur regarding valgus and varus deformity was conducted based on the patient’s age [16]. It was classified as physiological (angle within reference range) or as varus (angle below reference range) or valgus (angle above reference range) deformity.

Due to the neurocognitive impairment of the patients, clinical signs and symptoms such as pain and changes in walking patterns were reported by parents or caregivers. At the time of the radiological assessment, mobility was classified as unimpaired (no changes in known walking distance, walking time, and posture), impaired (restrictions in known walking distance or walking time but still able to bear weight and walk and/or exhibit postural asymmetry), or lost (reliant on a wheelchair).

All statistical analyses were performed using IBM SPSS Statistics for Windows, version 24.0 (IBM Corp., Armonk, NY, USA) and R statistical software (version 3.5.3). Baseline categorical variables are summarized using frequencies and percentages, and between-group comparisons were performed using Fisher’s exact test. Continuous variables are described as the mean ± standard deviation (SD) values, and Student’s *t*-test was applied for between-group comparisons. Paired data from the left and right hips were compared using paired *t*-tests for continuous data and the McNemar-Bowker test for categorical data.

Binary outcomes, e.g., osteonecrosis, pain, and walking ability, were analyzed using univariable logistic regression models applying Firth’s correction to the likelihood to account for the small sample size and the limited number of events. Bias-corrected odds ratios (ORs), 95% confidence intervals (CIs), and *p*-values are reported based on these models. The level of significance for all analyses was set at alpha = 0.05. Because this was an exploratory study, no adjustment for multiple testing was performed.

## Results

A total of 32 patients (21 males and 11 females) with a mean age of 11.0 years (SD 5.7, range 3.3–27.0 years) were enrolled in the study (Table 1). The mean age of male patients was 11.0 years (SD 5.4, range 3.3–23.6 years), and that of female patients was 11.0 years (SD 6.3, range 4.5–27.0 years). Eighty-one percent of patients presented with subtype MPS IIIA (*n* = 26); four patients had MPS IIIB and two MPS IIIC.

Genetic data were available for all but five patients (84%). Twenty-three patients (72%) were categorized as severe, seven (22%) as intermediate, and two (6%) as unknown; for the unknown category, neither mutation analysis nor the FPSS (due to insufficient clinical data) allowed further classification of the disease severity. No cases of the attenuated disease were identified in the study group.

**Fig. 2:**
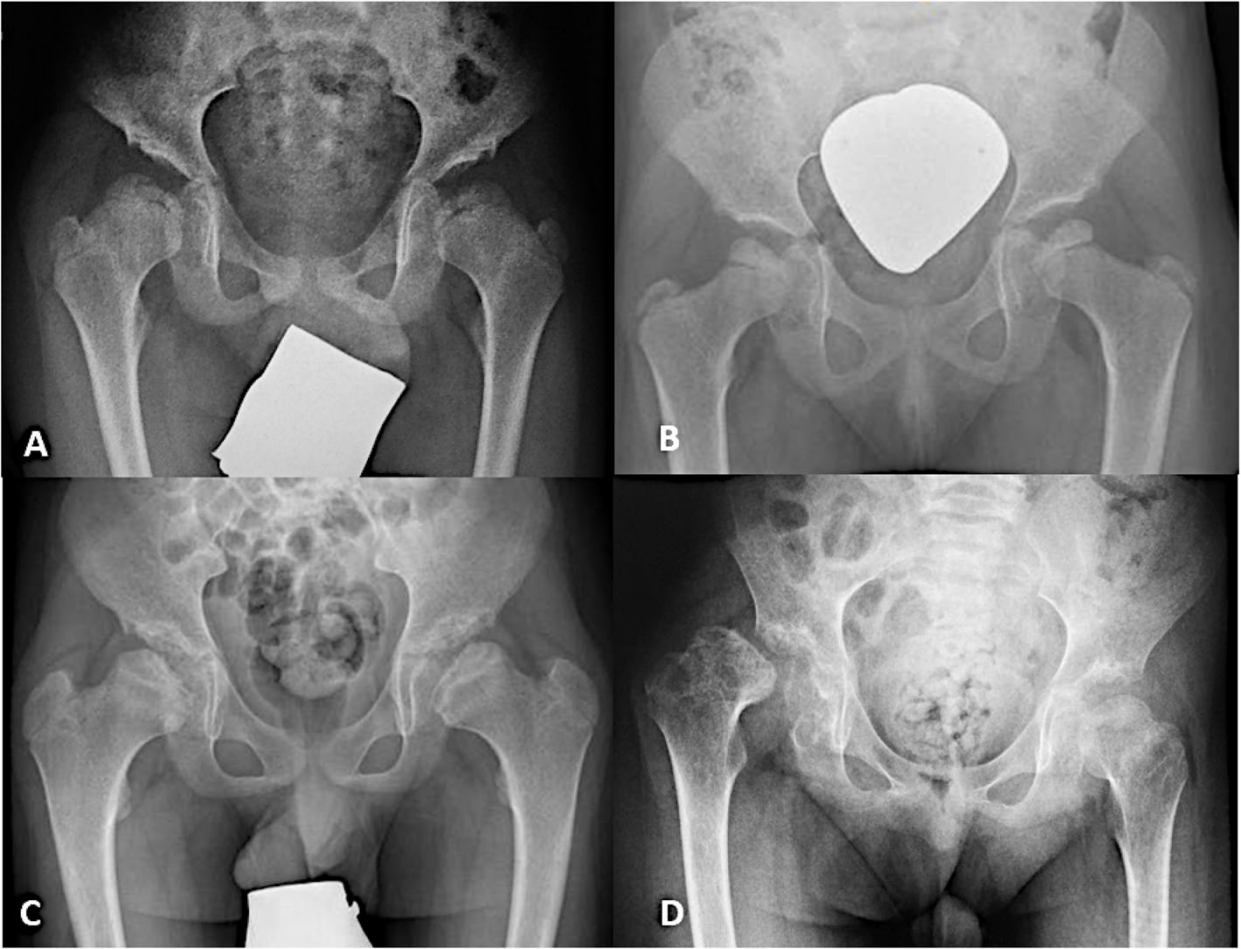
Presentation of osteonecrosis of the femoral head in severely affected MPS III patients. **a** An 8-year-old male MPS IIIA patient with severe dysplasia of both hips and osteonecrosis of both femoral heads. **b** An 8-year-old female MPS IIIA patient with physiological acetabular coverage, but osteonecrosis of the femoral head on both sides. **c** A 12-year-old male MPS IIIA patient with coxa vara and severely dysplastic acetabular coverage. **d** A 10-year-old male MPS IIIA patient with osteonecrosis of both femoral heads and dislocation of the right hip joint

Osteonecrosis of the femoral head was observed in 17 patients, and 13 out of these 17 patients were bilaterally affected (Table 2, Fig. 2). There was no statistically significant difference between the right and the left hips (*p* = 0.306). Fifty-nine percent of patients older than 10 years, 60% of patients from 5 to 10 years of age, and 40% of patients younger than 5 years of age had osteonecrosis of the head of the femur. Patients with a severe phenotype showed significantly higher rates of osteonecrosis (bias-corrected OR = 6.6, 95% CI [1.1–71], *p* = 0.036), and 82% (14/17) of patients with osteonecrosis exhibited a severe phenotype.

The mean CE angle was 25.3° (SD 13.4, range − 35.0 to 47.0°). A mean of 16% was found for MP (SD 17.9, range 0–100%). Dysplasia of at least one hip was documented in 28% of the patients (9/32, 4 females, 5 males). Four patients were affected unilaterally; 5 were affected bilaterally. All patients with hip dysplasia had a severe phenotype, compared to 14 of 23 patients without dysplasia and a severe phenotype (OR 9.8, 95% CI [0.99–1331], *p* = 0.052). Focusing on the single hips, a statistically significant association was evident between the presence of hip dysplasia and osteonecrosis of the femoral head (bias-corrected OR 3.5, 95% CI [1.1–13], *p* = 0.04). In contrast, osteonecrosis of the femoral head was documented in 43% of patients without dysplasia (10/23) (bias-corrected OR 3.9, 95% CI [0.8–24], *p* = 0.090). Dysplasia was found in only two patients of 15 without osteonecrosis of the femoral head.

One patient with osteonecrosis of the femoral head also had dislocation of the hip joint (Fig. 2). To alleviate the chronic pain of this patient, a salvage procedure with resection of the femoral head and angulation of the proximal femur was performed.

**Fig. 3:**
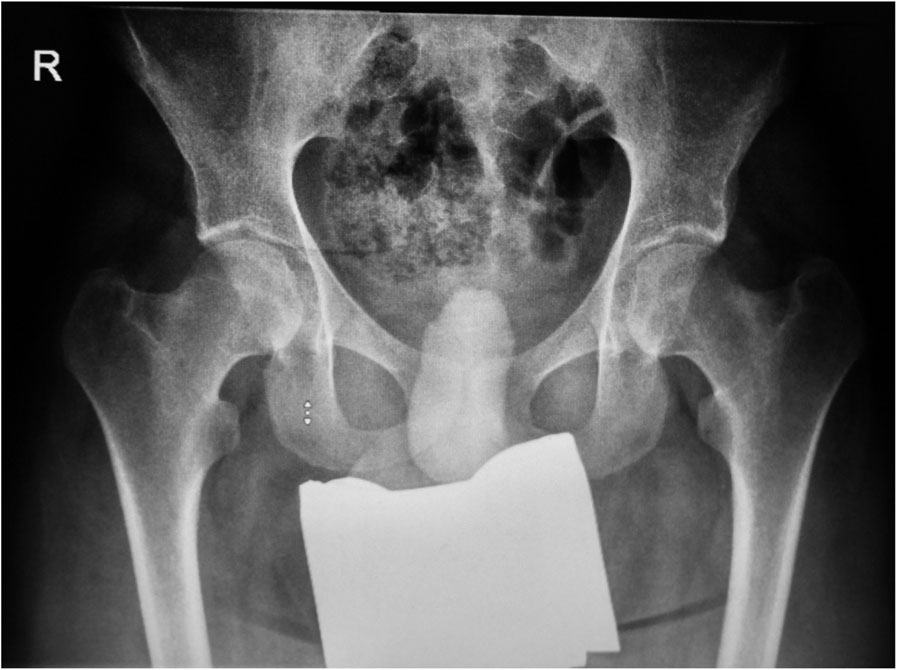
A 20-year-old MPS IIIA patient with an intermediate phenotype and physiological coverage of the femoral head. The neck of the femur is short and in a varus position. There are no radiographic signs of osteonecrosis of the femoral head

The anatomy of the femoral neck was described by the femoral neck-shaft angle, with a mean angle of 125.7° (SD 8.2, range 107–146°). In relation to the age of the patient, 53 of 64 hips were in a pathological varus position, and 8 hips were in valgus position; only 3 hips were physiological when compared to reference values (Fig. 3). No statistically significant correlation was observed between the femoral neck-shaft angle and osteonecrosis of the femoral head (OR of one degree increase 1.03, 95% CI [0.97–1.10], *p* = 0.312).

Due to the severe cognitive impairment of the patients, personal reports on pain were not collectable. Thus, parents or caregivers reported on the impression of pain in nine cases, and there was no evidence of pain in 21 patients. In two cases, the parents were unsure if their child suffered from pain. Changes in walking pattern or asymmetrical posture were documented for eight patients. Walking abilities were unimpaired in 14 patients and impaired in nine. In addition, nine patients were immobile and reliant on a wheelchair. In this group, seven patients had osteonecrosis of the femoral head of at least one hip, but no statistically significant association between mobility and osteonecrosis of the femoral head was identified (OR of osteonecrosis for impaired walking abilities 1.2, 95% CI [0.3–4.9], *p* = 0.760, OR for wheelchair reliance 3.9, 95% CI [0.8–24], *p* = 0.090). Moreover, pain was not a predictive indicator for osteonecrosis of the femoral head. Pain was reported in 29% (5/17) of patients with osteonecrosis only, compared to 4 of 15 patients (26%) without osteonecrosis (OR 1.1, 95% CI [0.3–5.2], *p* = 0.877). In the group of patients for whom radiographs were performed as a baseline assessment (patients without clinical symptoms), osteonecrosis was observed in 56% (9/16).

## Discussion

The present study describes hip pathologies and their influencing parameters in patients with MPS III. The high rate of osteonecrosis of the femoral head in the present study is in accordance with the literature [4, 5]. The study by de Ruijter et al. assessed 33 patients with MPS III and described signs of osteonecrosis of the femoral head (Ficat stage ≥ 1) in 24% [4]. The higher rate of 53% osteonecrosis in the current study can be explained by the lower incidence of intermediate and attenuated disease in the present study population versus the previous study population (intermediate/attenuated cases 22% versus 52%; severe cases 72% versus 45%). As no femoral head osteonecrosis in patients with an attenuated phenotype was observed, de Ruijter et al. concluded that disease severity appears to be a risk factor for femoral head osteonecrosis in MPS III patients. The present data is supporting this hypothesis. In addition, patients with a severe phenotype showed significantly higher rates of osteonecrosis of the femoral head compared to those with an intermediate phenotype.

Dysplasia of the hip is a common orthopedic pathology in MPS patients. Moreover, it is found in almost all patients with MPS I H (Hurler syndrome) and can also be present in MPS II, III, IV, and VI [5, 17–20]. In the literature, the rate of hip dysplasia in patients with MPS III varies between 18 and 44% [4, 5]. In the present study, the rate of hip dysplasia was 28% and was significantly associated with a high rate of osteonecrosis of the femoral head. Wang et al. described the same significant correlation in MPS IVA patients [20].

Considering that severely affected MPS III patients are at a higher risk of developing osteonecrosis, systemic influence besides mechanical deterioration needs to be discussed. Glycosaminoglycan (GAG) storage, which induces a complex sequence of molecular changes leading to inflammation, synovial hyperplasia, and cartilage apoptosis [8], is assumed to play a major role in joint and bone pathologies in MPS diseases. Animal and human studies investigating the treatment effects of anti-inflammatory drugs such as pentosan polysulfate (PPS) on skeletal pathologies in MPS III are under investigation [21]. In addition to inflammation, many studies in various MPS animal models have reported early abnormalities of chondrocyte organization in the growth plate and architecture of cortical bone which could be a trigger for abnormal bone modeling and remodeling leading to secondary hip deformities [22, 23]. In addition, Nur et al. found a 20% prevalence of low bone mineral density (BMD) and a 60% prevalence of vitamin D deficiency in MPS III patients [24]. Although these authors did not report osteonecrosis of the femoral head in the patients, there might be an association, as described earlier for other etiologies, such as alcoholic, steroid-induced, or idiopathic osteonecrosis [25].

**Fig. 4:**
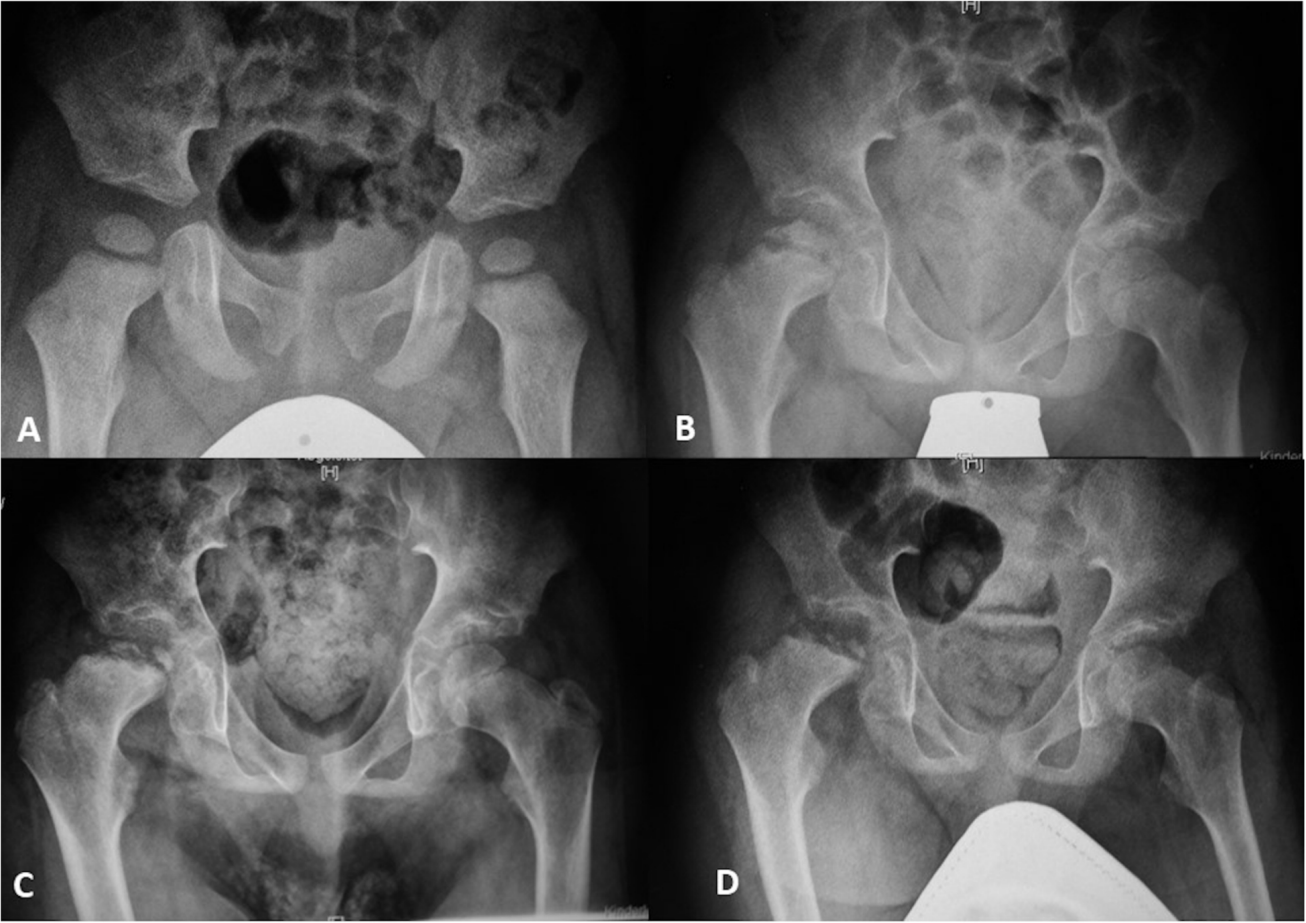
A male MPS IIIA patient with a severe phenotype. **a** At 3 years of age, there were no hip pathologies. **b** At 10 years of age, Perthes-like necrosis of the right hip was found; note the metaphyseal involvement and the condensation of the epiphysis. **c** Seven months later. **d** Eleven months after diagnosis

The radiological course of osteonecrosis of the femoral head in MPS III patients is in every aspect different than classical Perthes’ disease (Fig. 4) [5]. Further studies are necessary to address the question of whether the radiological changes in the femoral heads in MPS III patients comprise osteonecrosis due to avascularity or the substitution and remodeling of healthy bone due to the pathological storage of GAGs.

The limitations of this study are its retrospective design, the different indications for radiographic assessments and the absence of the lateral frog-leg view of the hips. Supine films might not be as reliable as standing radiographs in measuring the severity of dysplasia. Furthermore, due to the neurocognitive impairment of the patients, clinical signs such as pain were based on parents’ or caregivers’ observations, which might have resulted in underestimation of the true frequency of symptoms.

## Conclusions

The present study demonstrates a high rate of hip pathologies in MPS III patients. Hip dysplasia was seen in 28% of patients and was significantly correlated with femoral head osteonecrosis. Patients with a severe phenotype were significantly more affected by osteonecrosis of the femoral head (60.9%). Therefore, radiographs of the hips are highly recommended in routine baseline and follow-up assessments of patients with MPS III.

## Data Availability

The datasets used and analyzed during the current study are available from the corresponding author on reasonable request.

## Abbreviations

AP: Anteroposterior
BMD: Bone mineral density
CE: Wiberg’s center-edge
CI: Confidence intervals
FPSS: 4-Point scoring system
GAG: Glycosaminoglycan
HS: Heparan sulfate
MP: Reimer’s migration percentage
MPS: Mucopolysaccharidosis
MPS I H: Hurler syndrome
NAGLU: α-N-acetylglucosaminidase
OR: Odds ratios
PPS: Pentosan polysulfate
SD: Standard deviation
No.: Number
